# Analysis of the Chemical Composition and Morphological Characterization of Tissue Osseointegrated to a Dental Implant after 5 Years of Function

**DOI:** 10.3390/ijms23168882

**Published:** 2022-08-10

**Authors:** Josefa Alarcón Apablaza, Fernando José Días, Karina Godoy Sánchez, Pablo Navarro, Camila Venegas, Ramón Fuentes

**Affiliations:** 1 Research Centre in Dental Sciences (CICO-UFRO), Dental School—Facultad de Odontología, Universidad de La Frontera, Temuco 4780000, Chile; 2 Department of Integral Adults Dentistry, Dental School—Facultad de Odontología, Universidad de La Frontera, Temuco 4780000, Chile; 3 Oral Biology Research Centre (CIBO-UFRO), Dental School—Facultad de Odontología, Universidad de La Frontera, Temuco 4780000, Chile; 4 Scientific and Technological Bioresource Nucleus (BIOREN-UFRO), Universidad de La Frontera, Temuco 4780000, Chile; 5 Universidad Autónoma de Chile, Temuco 4780000, Chile; 6 Program of Master in Dental Science, Dental School—Facultad de Odontología, Universidad de La Frontera, Temuco 4780000, Chile

**Keywords:** bone regeneration, dental implants, biocompatible materials, microscopy

## Abstract

Osseointegration implies the coexistence of a biocompatible implant subjected to masticatory loads and living bone tissue adhered to its surface; this interaction is a critical process for the success of implants. The objective of this work is to analyze the osseoformation and osseointegration of a dental implant in operation for 5 years microscopically through morphological analysis of the surface and chemical composition through a variable pressure scanning electron microscope (VP-SEM) and energy dispersive X-ray spectrometry (EDX). The chemical composition and general characteristics of the structural morphology of random areas of the surfaces of an osseointegrated dental implant from an ex vivo sample were analyzed. On the surface of the implant free of bone tissue, titanium (TI) was mainly identified in the area of the implant threads and carbon (C) in the depth of the implant threads. Phosphorus (P), calcium (Ca), oxygen (O), carbon (C), with dense and homogeneous distribution, and, to a lesser extent, sodium (Na) were detected on the bone surface around the contour of the implant. Regarding the morphological characteristics of the implant surface, a rough structure with some irregularities and detachments of the implant lodged in the bone tissue was observed. Microscopic analysis showed calcified bone tissue distributed in an orderly manner on the coronal and medial surface and sinuous and irregular in the apical area, with the presence of red blood cells. The composition of the implant allows a dynamic process of bone remodeling and regeneration subject to the biological and mechanical needs of the operation. Dental implants are shown to have exceptional and long-lasting biocompatibility that enables the formation of mature peri-implant bone tissue.

## 1. Introduction

Bone tissue is a specialized connective tissue made up of inorganic (~70%) and organic (~30%) fractions. The main inorganic component is hydroxyapatite (HA) Ca10(PO4)6(OH)2, a mineral composed of calcium phosphate. The organic fraction consists mainly of type I collagen fibers, in addition to specialized bone cells, including osteoclasts, osteoblasts, and osteocytes, which allow the bone to be constantly changed by remodeling and repair processes [1,2]

A dental implant is a biomaterial device inserted into the maxillary bone to replace the root of a missing tooth [3]. The placement of dental implants in partially or totally edentulous patients has become a routine treatment for oral rehabilitation. Different materials commonly used in the manufacture of dental implants have been documented. Titanium (Ti) has been the first choice as a material for the manufacture of dental implants due to the combination of its outstanding characteristics, such as high resistance to fatigue, high resistance to corrosion and wear, and biocompatibility [4]. However, despite more than four decades of extensive research and commercialization of Ti dental implants, the biological mechanisms responsible for the osseointegration process of Ti are still poorly understood. Bone healing surrounding implants remains a complicated and dynamic physiological process, regulated by growth and differentiation factors released by blood cells and activated at the bone–implant interface to achieve osseointegration [4,5].

Osseointegration is the process resulting from a direct structural and functional connection between the bone and the surface of the implant during functional loading, thus providing the basis for the desired function of the dental implant [3,4,5,6,7]. The osseointegration process of an implant in healthy conditions is complex and takes several weeks to heal. Immediately after implantation, inflammatory cell and bone cell reactions take place at the bone–implant interface. These events are followed by the process of bone regeneration, which is regulated by various biological factors in the proximities of the implant. Subsequently, bone mineralization occurs at the contact and distance sites of the dental implants [8].

The speed and quality of osseointegration in Ti implants are related to their surface properties. The surface composition and roughness are parameters that play a fundamental role in osseointegration [9]. A Variable Pressure Scanning Electron Microscopy (VP-SEM) is capable of analyzing the micromorphology of the mineralized tissue in the sample and can produce attractive results in the investigation of the peri-implant-bone interface. Energy-dispersive X-ray spectroscopy (EDX) is useful for studying the elemental composition of mineralized tissues or mineral deposits (apatite). In addition, it can detect elements that may have migrated from an implant material to the bone tissue [10].

Animal experiments have been used to test the osseointegration of dental implants [8,11]. However, specific animal models that resemble human medical conditions are needed, in which responses to biomaterials can be tested before clinical use [8,10]. This is why for accurate judgment, the results of careful experimental characterization of osseointegration obtained from a recovered human implant model may be favorable for proper clinical translation.

Therefore, the best way to assess osseointegration is to retrieve an osseointegrated implant from native human bone and analyze the bone–implant interface microscopically [4]. For this reason, this study aims to analyze the microsurfaces of an implant and associated bone tissue with 5 years of operation under VP-SEM and the elemental composition of the implant surfaces and attached bone by EDX system of an implant dental lost due to the fracture of the head of the implant. There are no records of VPSEM and EDX that analyze a human dental implant with years of function.

## 2. Results

### 2.1. Case Report

A sample of Dentium^®^ dental implant 3.6 mm platform and 3.4 mm body was obtained, in clinical operation for 5 years, from a 55-year-old male patient, complete dentition, healthy, non-smoker. The analyzed implant was extracted due to a fracture of the implant head, which prevented the prosthetic connection. The implant was extracted using a trephine drill with a 4 mm internal diameter.

Informed consent was obtained from the subject prior to the study. Patient data, including the indication for implant removal, were collected through the electronic medical record system.

### 2.2. Surface Morphological Analysis of Osseointegrated Dental Implants

#### 2.2.1. Morphological Analysis of the Dental Implant Surface

**Figure 1:**
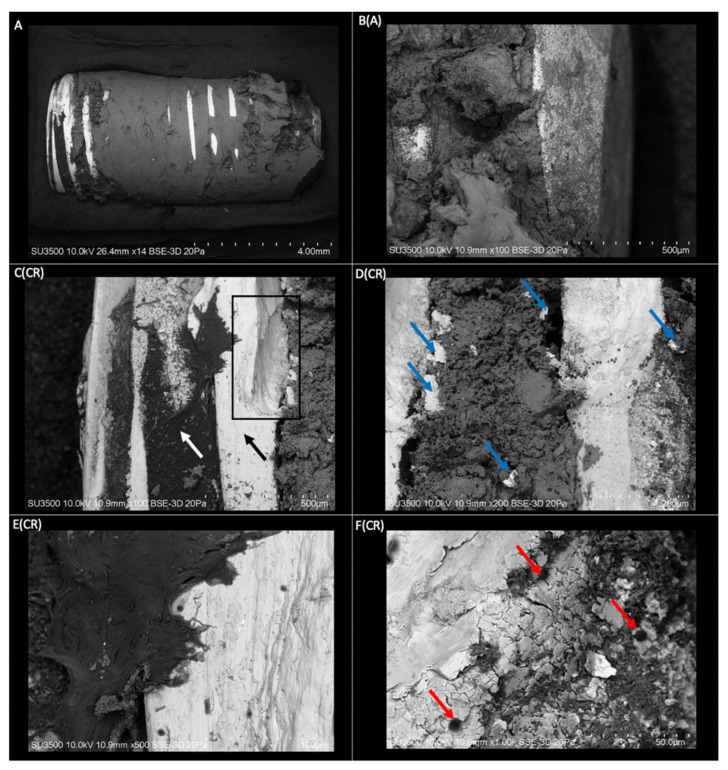
(**A**) Three-dimensional structure of a dental implant with mature bone tissue obtained by VP-SEM (Mag: ×14). **B(A)** Apical view of the implant structure with an integral union of mature bone tissue (Mag: ×100). **C(CR)** Coronal area of the implant with threads free of bone tissue (black arrow), and depth of the threads with the presence of organic tissue (white arrow). Area of the implant threads with a defect in its structure (rectangle) (Mag: ×100). **D(CR)** Coronal view of the implant with an integral union of bone tissue between the threads. Spalling of the implant surface lodged in the bone tissue (blue arrows) (Mag: ×200). **E(CR)** Coronal area thread of the implant with a higher magnification. A rough structure with linear irregularities is displayed in orientation to the implant threads (Mag: ×500). **F(CR)** View of a thread with higher magnification. Visualization of roughness on the implant surface. Presence of bone tissue penetrates the roughness and blood cells on the surface of the implant (red arrows) (Mag: ×1000).

The dental implant sample with integrated bone tissue processed for observation under a scanning electron microscope is shown in Figure 1 at different magnifications. The light/white areas of the images represent the dental implant, while the darker areas represent the bone tissue. The implant is practically surrounded by bone tissue integrated into the metallic surface. The implant surface without bone tissue is the most pronounced area of the threads and the coronal area of the dental implant. At higher magnification, the exposed surface of the implant can be seen with the presence of linear irregularities in orientation to the thread (Figure 1E(CR)) and roughness that favors the union and penetration of bone tissue through the surface of the dental implant (Figure 1F(CR)). An irregular surface characterized by surface scaling (Figure 1D(CR),F(CR)) and an undercut in the structure Figure 1C(CR) may be associated with implant removal with a trephine.

#### 2.2.2. Superficial Morphological Analysis of Bone Tissue Associated with Dental Implants

**Figure 2:**
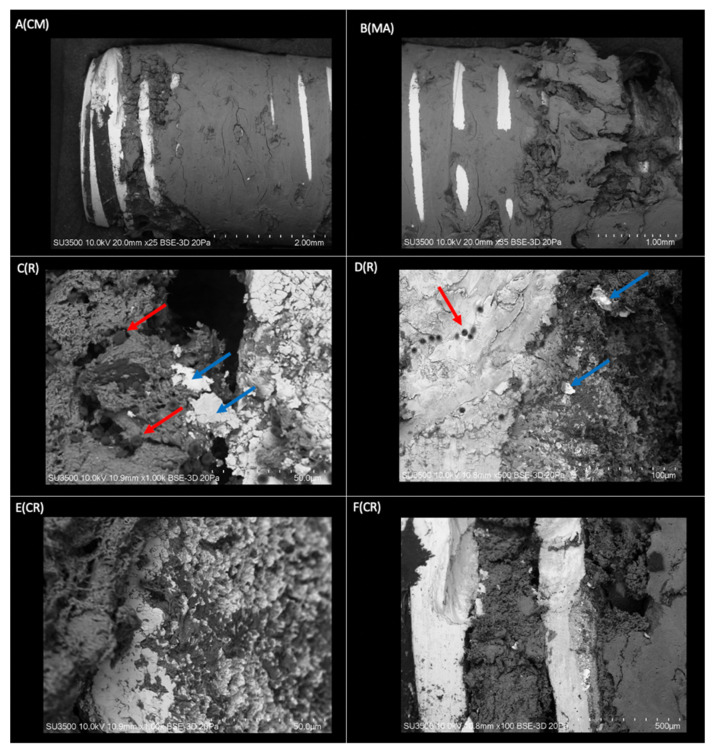
Three-dimensional structure of the implant sample covered with bone tissue obtained by VP-SEM. **A(CM)** Coronal area-middle of the implant. The bone tissue homogeneously covers most of the implant. Smooth surface in conformation to bone laminar zones (Mag: ×25). **B(MA)** Mid-apical area of the dental implant. Morphologically rougher and more sinuous tissue in the apical sector compared to the middle zone (Mag: ×35). **C(R)** Thread on the right side of the image and groove on the left side of the image, covered by mineralized and non-mineralized cancellous bone tissue with associated red blood cells (red arrows) and flaking of the implant surface (blue arrow) (Mag: ×1000). **D(R)** Implant thread (light grey) with fully associated bone tissue (dark grey) and presence of red blood cells (red arrow). Spalling of the dental implant surface (blue arrow) (Mag: ×500). **E(CR)** View of an implant thread at higher magnification (light gray area). Integration of bone tissue (dark gray area) in the irregularities on the implant surface (Mag: ×1000). **F(CR)** Implant threads without bone tissue and grooves completely covered by bone tissue (Mag: ×100).

The sample of bone tissue integrated into a dental implant, processed for observation under a VP-SEM, is shown in Figure 2. The evaluation confirmed that the implant is surrounded by bone tissue integrated into the metallic surface that joins the implant threads (Figure 2A(CM),B(MA)). This confirms that practically the entire implant surface is involved in the osseointegration process, showing a very close spatial relationship between the Ti and bone tissue in the irregularities of the implant surface (Figure 2E(CR)). In a lower magnification image (Figure 2B(MA)), bone tissue can be seen crossing the areas of the metallic crests of the threads. At higher magnification (Figure 2F(CR)), the osseointegration process is again confirmed by the presence of bone tissue occupying the entire slot between the implant threads. The coronal–medial area of the implant shows a compact and homogeneous bone structure; however, in the apical zone, it is characterized by a more irregular and sinuous morphology. In the threads of the coronal area, the structure of the implant can be observed, which indicates that the space between the cell layer and the oxide is very thin, and there is bone resorption at the level of the coronal third. The presence of mineralized bone tissue interface with small groups of erythrocytes is an indicator of the maturity of the osseointegration process.

### 2.3. Chemical Analysis of Dental Implants and Bone Tissue

**Figure 3:**
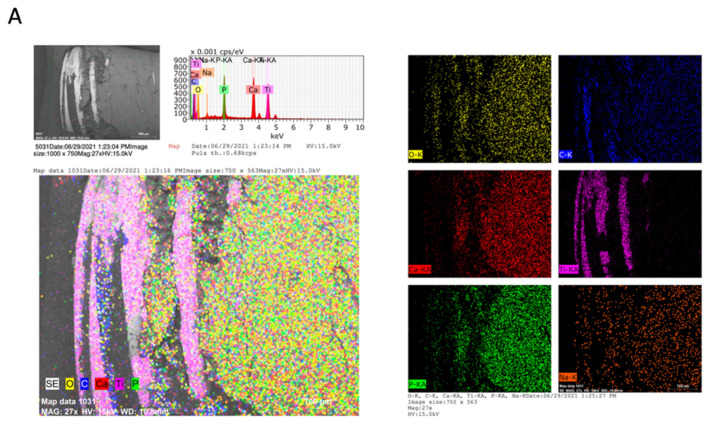

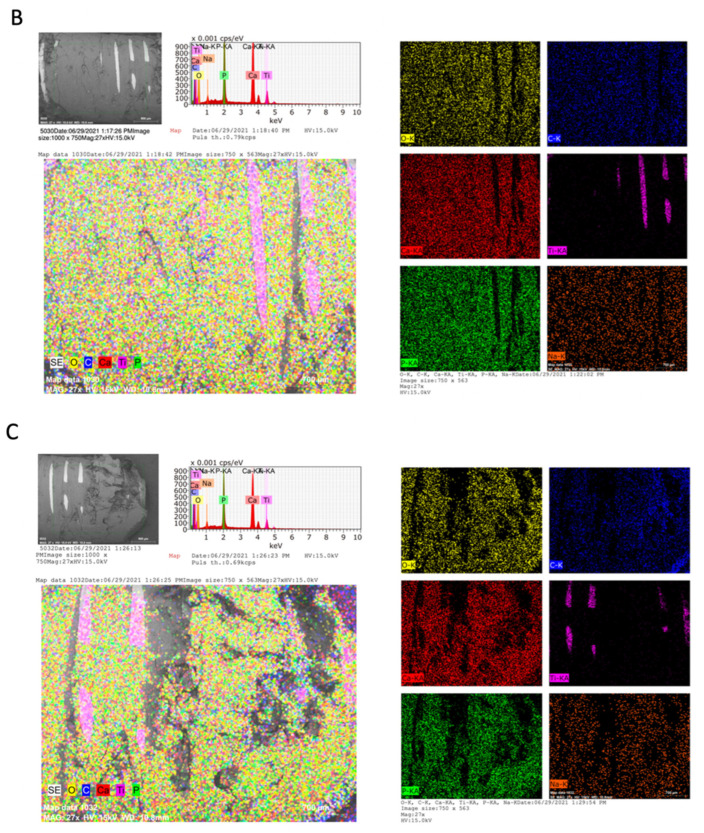
Micrographs (SEM-EDX) of the dental implant with integrated bone tissue of the (**A**) Coronal, (**B**) Medial, and (**C**) Apical areas. Colorimetry graph with the density of chemical elements. Yellow-oxygen (O); Blue-carbon (C); Red-calcium (Ca); Pink-titanium (Ti); green-phosphorus (P); Orange-sodium (Na). The EDX spectrum is also shown with peaks for the identified elements.

The EDX method included qualitative microanalysis as well as element distribution mapping. Figure 3 provides the chemical information of the surfaces through elemental mapping by specific colors for each element. A similar qualitative composition was observed on the implant surfaces in all the areas analyzed. On the implant surface, TI was identified as the main element, in the area of the threads and coronal area of the implant, with a homogeneous distribution on the implant surface. On the other hand, on the surface of the bone tissue that surrounds the dental implant, a qualitative composition was identified in a high proportion of Ca, P, O, and, to a lesser extent, Na and C in uniform distribution on the surface. C is the main organic component; it was identified in a greater proportion in the depth of the implant threads and a smaller quantity on the implant surface. The areas with higher concentrations of C showed a lower concentration of Ca and P. In addition, Na content was identified in a lower proportion on the bone tissue surface. The sinuous zones of greater depth, mainly in the apical zone, are not able to be analyzed by EDX since the analysis captures a depth of 10 to 15 microns. 

## 3. Discussion

The present study analyzed the surface of an implant and associated bone tissue with 5 years of operation under VP-SEM and the elemental composition of the implant and bone surfaces by EDX. This analysis will provide basic information to understand an implant’s osseointegration process during a long operation period. The surface topography of the implants, i.e., the roughness and the orientation of these surface irregularities, constitutes a research challenge and interest for oral implantology since they are of the utmost importance to obtain successful clinical results and provide better molecular absorption. It has been widely described that an isotropic implant surface, i.e., with roughness or microcavities, fissures, or cracks, can favor the union between the implant surface and bone, increasing the integration of the implant compared to minimally rough surfaces [12,13]. Thalji, G, et al. identified the first molecular processes involved in osseointegration associated with the nanosurface of featured implants. The presence of nanosurface features modulated the bone response in vivo. Gene regulation involving osteogenesis, as well as inflammatory/immune responses that occur based on surface topography, can affect bone mass shortly after implant placement [14].

Surface roughness can be divided into three levels depending on the scale of the features: macro, micro, and nano-sized topologies [15]. The macro-level is for topographical features defined as being in the range of millimeters to tens of microns. This scale is directly related to the geometry of the implant [9,16]. Morphology at this level has been shown to improve mechanical integration between the implant surface and surrounding bone, improving primary implant fixation and long-term mechanical stability [16]. In this study, it was observed that the depth of the implant grooves achieved successful long-term osseointegration. The micrometric range of dental implants is defined by the roughness of the implant surface [16]. This study demonstrates on higher zoom images that this range of roughness maximized the interlock between the mineralized bone and the implant surface and, therefore, greater bone-to-implant contact compared to smooth surfaces [9]. Finally, surface profiles in the nanometer range play an important role in protein adsorption, osteoblast cell adhesion, and thus the rate of osseointegration. Various methods have been developed to create these ridges [9]. This biological and physical response has corresponded with the results of this study in images with higher magnification, demonstrating an intimate integration of bone tissue in the depths of the grooves and irregularities of the implant surface. The sample demonstrated a high level of direct bone-to-implant contact. In contrast, the implant threads with a smooth surface without major irregularities were observed to be free of bone tissue. Therefore, we can deduce that the surface of the implants has a significant influence on osseointegration. 

In the results of this study, homogeneous bone remodeling can be described in the coronal and middle surface of the implant with mainly cortical characteristics, where the arrangement of circumferential laminae was identified. However, in the apical third, a sinuous adaptive bone remodeling was observed, irregular and discontinuous. This bone formation can be associated with mechanical demands according to the distribution of occlusal loads, which can alter its configuration. Compressions can generate bone growth with deformations in the macrostructure since bone tissue can adapt to its biological and mechanical environment, modifying its structure depending on loads [17,18,19]. This process is known as bone remodeling, which locates the parts where it is necessary to remove, replace or increase their mechanically functional bone architecture. This bone remodeling responds to the intensity of the stimulus associated with applied mechanical loads [20]. In addition to the formation of sinuous bone tissue in the apical area, in this study, we can associate the significant occlusal load with the marginal bone resorption of the implant at the level of the coronal third. Studies have related the exposure of the coronal surface of the implant to the supra-crestal connective tissue compartment due to occlusal loads for more than one year [21]. We can observe this in this study with the exposed threads of the implant and the presence of connective tissue on the surface of the coronal third of the implant.

The application of chemical element mapping is useful to demonstrate how the distribution of elements on the sample surface is affected. The chemical composition of the implant analyzed in this study showed mostly the presence of Ti, using EDX and VP-SEM. In general, dental implants are made of commercially pure Ti due to their proven biocompatibility [22,23]. On the other hand, O was found in a lower proportion. The presence of O on the surface suggests the formation of titanium oxide on the implant surface. The biocompatibility of Ti is related to the properties of its surface oxide. In contact with air or water, Ti rapidly forms an oxide thickness of 3–5 nm at room temperature. The most frequent oxide is titanium dioxide (TiO_2_). This oxide is very resistant to chemical attack, which makes Ti one of the most corrosion-resistant metals [24,25,26]. The oxide surface has a negative charge at physiological pH. It has been suggested that this promotes the formation of an amorphous Ca compound on the implant surface, which acts as a precursor for subsequent apatite nucleation. More generally, a biochemical link at the bone–implant interface has been proposed to improve osseointegration properties [27]. 

The results of the chemical composition of bone tissue described above correspond to these results [28,29,30,31]. It presents mostly elements such as Ca, P, O, C and Na. Bone is a mineralized connective tissue formed by osteoblasts that deposit collagen and release Ca, magnesium (Mg), and phosphate ions that chemically combine within the collagen matrix into a crystalline mineral known as bone HA. On average, bone tissue contains about 10–25% water, 25% protein fibers such as collagen, and 50% HA [32]. Thus, Ca, P, and O are bulk inorganic bone components of normal human bone tissue that were found to be present in the bone tissue for the formation of HA. This is a calcium phosphate hydrated from the mineral group of apatite with the chemical formula Ca_10_(PO_4_)_6_(OH)_2_ and a Ca/P ratio of 1.67 [33]. In addition, other elements such as C were found on the surface of the bone tissue and the implant grooves [25]. C is the main organic component, and the biological apatite crystal lattice contains carbonate [33]. It has been shown in a study that areas with a high accumulation of Ca and P present less C [34]; this suggests that the concentration of organic components decreases while mineral components increase during bone development [35], i.e., the matrix of collagen becomes more mineralized [36]. This study shows that Ca and P are present in large amounts in the bone matrix and coincide with each other in quantity, while C is located in connective tissue in the coronal third and in lesser amounts in bone tissue, which suggests the presence of bone tissue with high mineralization. Finally, Na was also present in the bone tissue in a smaller proportion. It has been shown that among the biological functions of bone tissue is the deposit of minerals, where sodium residing in bone tissue stands out [37,38].

The present study has certain limitations; it is about the presentation of an alone case report; therefore, it would be advisable to evaluate a larger number of samples to draw more significant conclusions about the osseointegration of implants ex vivo after years of operation, analyzing the surface of the dental implant in its nano, micro, and macro structure and evaluating how osseointegration, mineralization, and bone remodeling vary over the years of operation. 

## 4. Materials and Methods

### 4.1. Case Report

This paper was written in accordance with the CAse REport (CARE) guidelines (https://www.care-statement.org) (accessed on 6 June 2022)

### 4.2. Surface Morphological Analysis of Osseointegrated Dental Implants

**Figure 4:**
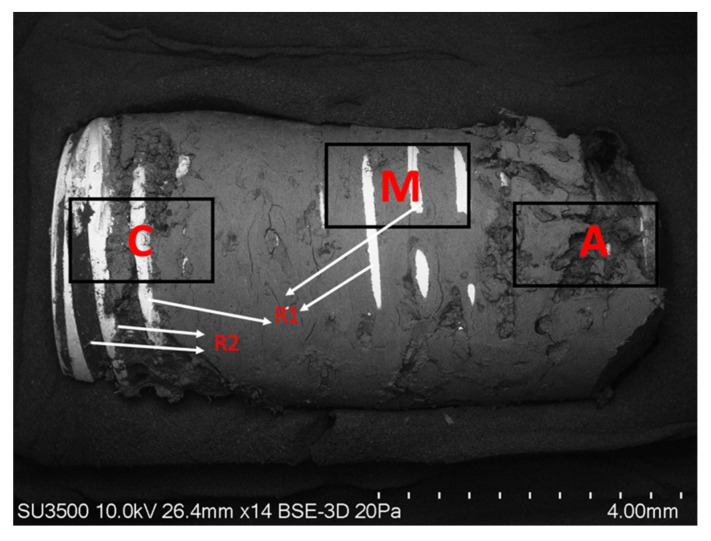
SEM photograph showing the areas of the implant examined by VPSEM. (C) Coronal area of the implant with osseointegrated bone tissue; (R) implant threads (R1) zone free of bone tissue in the pronounced areas of the width of the tread (R2) presence of osseointegrated bone tissue in the depth of the thread. (M) Mesial area of the implant (A) Applicable area of the implant (Mag: ×14).

A three-dimensional morphological analysis was performed with VPSEM. The implant and integrated bone tissue samples collected during the resection surgery were fixed on the metal support with the help of double-sided carbon tape. Random areas with representative features were chosen for the micrograph. The points analyzed by VPSEM are classified according to their distribution (R: thread, A: the apical third of the implant, M: the middle third of the implant, C: the coronal third of the implant) (Figure 4). The SEM was obtained at an acceleration potential of 10.0 kV and a variable pressure of 20 Pa. The magnifications of the ×14, ×20, ×35, ×100, and ×200 photomicrographs were standardized, while in cases of specific regions of interest, ×500 and ×1000 images were also obtained. The descriptive analysis of the morphological characteristics of the surface was based mainly on surface defects, roughness, and irregularities. For its part, the morphological analysis of bone tissue was based on the presence of cells, characterization of the bone surface, irregularities, bone growth patterns, and bone integration in the implant surface.

### 4.3. Chemical Analysis of Osseointegrated Dental Implants

Elemental semi-quantitative analysis was carried out on the implant sample with bone tissue by EDX using an X 410-M flash detector (Bruker, Berlin, Germany) associated with VPSEM. This detector captures the energy from the X-rays generated on the surface, which are characteristic of each element in the sample, which is why they provide us with information on the elemental composition.

**Figure 5:**
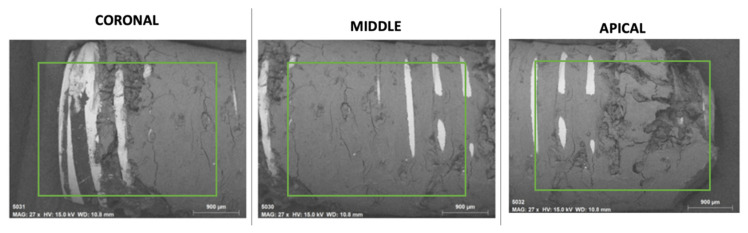
Semi-quantitative elemental quantification-(EDX Method) of the coronal A(C), medial B(M), and apical C(A) areas of the dental implant covered by bone tissue.

The EDX method involved a semi-quantitative microanalysis in different areas of the sample, including the mapping of element distribution in the areas of the implant free of bone tissue and areas of the dental implant covered by bone tissue. Representative areas of the sample were analyzed under Mag: 27× magnification and acceleration potential of 15 kV. The depth of analysis was estimated at 10 microns. The analysis of the areas of the implant yielded a map with the different elements that the selected area of the sample possesses. On distribution maps, relative concentrations were indicated by color density. The sample was connected to the metal support using double-sided carbon tape. All the chemical elements detected in the samples were analyzed, and comparisons were made in different areas of the sample of the coronal (C), middle (M), apical (A) zone, and zone of the exposed implant threads without bone tissue (R) (Figure 5).

## 5. Conclusions

This study analyzed the surfaces of an implant and associated bone tissue with 5 years of operation under VP-SEM and the elemental composition of the surfaces using EDX. The analyzed composition of the implant surface mostly showed the presence of Ti elements biocompatible with the organism and the Ca, P, O, C, and Na in the bone tissue. The composition of the implant allows a dynamic process of bone remodeling and osseointegration subject to the biological and mechanical needs of the operation. Dental implants are shown to have exceptional and long-lasting biocompatibility that allows for the formation of mature peri-implant bone tissue.

## Data Availability

Not applicable.
